# Exploring the benefits of inoculated cowpeas under different climatic conditions in Namibia

**DOI:** 10.1038/s41598-023-38949-2

**Published:** 2023-07-20

**Authors:** Livia Rasche, Joscha N. Becker, Percy Chimwamurombe, Annette Eschenbach, Alexander Gröngröft, Jihye Jeong, Jona Luther-Mosebach, Barbara Reinhold-Hurek, Abhijit Sarkar, Uwe A. Schneider

**Affiliations:** 1 Research Unit Sustainability and Climate Risks, Universität Hamburg, Grindelberg 5, 20144 Hamburg, Germany; 2 Institute of Soil Science, Universität Hamburg, Allende-Platz 2, 20146 Hamburg, Germany; 3 Department of Natural and Applied Sciences, Namibia University of Science and Technology, Brahms St, Windhoek, Namibia; 4 Research Group Molecular Plant-Microbe Interactions, University of Bremen, Loebener Str. 5, 28359 Bremen, Germany

**Keywords:** Microbiology techniques, Rhizobial symbiosis, Climate-change impacts, Climate-change adaptation, Agroecology

## Abstract

Cowpeas (*Vigna uniculata* L. Walp) are grown by many smallholder farmers in sub-Saharan Africa for food and their ability to fix nitrogen even under stress. Their performance depends on the indigenous rhizobial strains that live in symbiotic association with the roots; it can be enhanced if the seeds are inoculated with more effective ones. Data of the effectiveness of the technique under a variety of climatic conditions is rare. Here, we thus use a model to upscale two field experiments conducted in Namibia to include different climate change scenarios. The simulations show that non-inoculated cowpeas have mean yields of 0.5 t/ha and inoculated cowpeas 1 t/ha. If climatic conditions are favorable (cool and wet), estimated yield differences increase to over 1 t/ha. In dry years (< 200 mm), the average yield difference is only 0.1 t/ha. In the far future (2080–2100), instances of dry and hot years will increase. Using inoculated cowpea seeds instead of non-inoculated ones thus does not benefit farmers as much then as in the near future (2030–2050). In conclusion, using cowpea seeds inoculated with an efficient rhizobial strain can significantly increase yields under varying climatic conditions, but yield advantages decrease markedly in very dry and hot years.

## Introduction

Cowpea (*Vigna unguiculata* L. Walp) is an important protein crop to secure domestic food production in the semiarid parts of sub-Saharan Africa where other food legumes can struggle to thrive^1,2^. In 2020, cowpeas were produced on nearly 14 million ha of land in the central and southern parts of the African continent (https://www.fao.org/faostat/en/#data). The versatile crop can produce grain and stover for food and forage^3^ and fix atmospheric nitrogen even under water or temperature stress^4^. Under optimal conditions, cowpeas can fix up to 240 kg N ha^−1^^5^, which makes them a good choice for smallholder farmers struggling to procure mineral nitrogen fertilizer^6^.

The efficiency of nitrogen fixation depends on the cowpea variety and the rhizobial strains that live in symbiotic association with the roots of the cowpea^5,7^. In some regions, local rhizobia can be less effective (e.g.^8,9^). They may be low in population or are not efficient in N_2_ fixation^10^. Soil nutrient deficiency can also contribute to less nodulation by local strains^11^. Additionally, high temperatures and water scarcity can indirectly affect successful root nodulation by reducing rhizobial mobility or leading to desiccation and cell death^12^. In these cases, inoculation with previously isolated, more effective rhizobial strains can increase nitrogen fixation and yields^13^. Inoculants may also protect cowpeas from parasitic plants and phytopathogens^14,15^ and enhance the content of bioactive compounds in the yield, which increases the nutritional value^16^. They can solubilize phosphate^17^, supply growth-promoting hormones to host plants^18^, and improve the resistance of cowpeas to water stress^19^.

Many benefits associated with inoculation have been studied in small-scale greenhouse and field experiments. The scope of these experiments is often limited to one growing season and few treatments due to space, infrastructure, and funding constraints. In this paper, we use a biogeophysical crop model to upscale the results of a small-scale field trial conducted in 2017/2018 in the Northern region of Namibia. In this trial, cowpeas were grown with and without *Bradyrhizobium* inoculation and their yields and other crop parameters were compared. As the growing season was very dry, emergency irrigation was applied to avoid the loss of too many plants. In this upscaling study, we want to use the data collected in this trial as a baseline to answer further questions: How will inoculation affect yields under different climatic conditions? Can inoculated cowpeas withstand water stress better than non-inoculated plants? And lastly, how will the benefits of inoculated cowpeas change in the future?

## Material and methods

### Field experiments

The field trials were located at Ogongo (−17°40′12′′, 15°18′0′′, elevation 1111 m MSL) and Mashare (−17°53′24′′, 20°10′48″, elevation 1050 m MSL) in the Northern and North-eastern regions of Namibia. Each field measured 41 × 41 m, with 0.5 m fringe, 12 m per treatment block, and two meters separating the blocks. The study was laid out in a block design with four replications of each treatment. The treatments consisted of (i) cowpea without inoculant, (ii) cowpea without inoculant plus urea fertilizer, (iii) cowpea with inoculant. The cowpea variety Lutembwe from Zambia was used. In the treatment with inoculation, a strain of *Bradyrhizobium* that was previously collected in the Kavango region (*Bradyrhizobium* strain 1–7)^11,20^ was added as inoculant to the cowpea seeds with peat as carrier. Bradyrhizobium sp. strain 1–7 effectively nodulates cowpea and Bambara groundnut^11^. It is a heat-resistant symbiont, as it showed growth still at 35–38 °C^11,20^ and is therefore likely to survive even under climate change.

Prior to planting, five soil samples were taken in each plot and mixed to yield one sample per plot. In Mashare, the planting was conducted on January 5 2018, and in Ogongo on February 2 2018. A pinch of phosphate fertilizer was added to every subplot, amounting to approximately 15 kg per hectare. In the urea-treatment, approximately 1.7 g of urea was added to every planting hole. During the growing season, irrigation water was applied once a week if conditions were dry. Single amounts ranged between 10 and 50 mm per week, up to 250 mm in total. The cowpeas were harvested on April 26 2018 in Mashare, and May 18 in Ogongo. After harvesting, the shoot wet weight and dry weight (g), the plant dry matter yield (kg/ha), the root wet and dry weight (g), the root dry matter yield (kg/ha), the total grain yield (g), the grain yield per area (kg/ha), the ten plants pod number, the average pod number per subplot and the 40 pods seed number were determined for each subplot as well as the number of harvested plants.

We calibrated the simulated grain yields of the crop model for two treatments: (i) Lutembwe cowpea without inoculant and (ii) Lutembwe cowpea with inoculant. We chose to omit the urea-treatment from the simulation experiment for two reasons: (1) In a survey among smallholders farmers of the region, which was undertaken in parallel to the field experiments, only two of the 97 participants reported that they used fertilizer on their cowpeas. We concluded that this was the general practice for the region and should serve as the benchmark for comparisons. (2) The results of the field experiments were inconclusive for the urea treatment. Non-inoculated cowpeas with urea fertilizer treatment had lower mean yields than non-inoculated cowpeas without urea fertilizer in Mashare, 1.3 t/ha (urea) vs. 2.0 t/ha (no urea), probably due to initial inhibition of nodulation and leaching from the sandy soil. We were unable to reproduce this effect with EPIC.

### Crop model

For the study, we used the Environmental Policy Integrated Climate (EPIC) model^21^. EPIC is a field-level biophysical process-based model which can simulate crop growth and crop yield, soil nutrient cycling, soil erosion, and different tillage and management practices. Crops are described by a unique set of crop parameters which include, e.g., radiation use efficiency, maximum potential harvest index, base temperature, optimal temperature, maximum potential LAI, maximum stomatal conductance, maximum crop height and root depth, and nitrogen, phosphorous and potassium uptake parameters. The plant biophysical processes simulated by EPIC include interception of photosynthetically active solar radiation dependent on LAI, conversion to biomass based on radiation use efficiency and crop growth stresses (nutrient and water availability, temperature), partitioning of the daily biomass increase into the root and aboveground biomass, and adaption of the harvest index to drought conditions^22^. EPIC has been used for many years to study different aspects of agricultural systems, such as yield gaps^23–25^, climate change impacts on crop yields^26^, environmental impacts^27,28^, soil degradation^29^, or soil erosion and nutrient leaching^30^.

### Model setup for calibration

The input data required for a simulation with the crop model EPIC consists of site data (location, elevation), daily weather data (temperature, precipitation, solar radiation, wind speed, relative humidity), soil data, and management data (dates for plowing, planting, harvesting, fertilizing, amount and type of fertilizer, seeding density). Site and management data were taken from the description of the field experiments. Daily weather data for the years 2017 and 2018 was downloaded from SASSCAL WeatherNet for the stations Mashare and Ogongo (https://www.sasscalweathernet.org). The soil input files were prepared based on the analysis of the soil samples taken prior to planting in 2018 (Table 1).

**Table 1:** Soil input data for Mashare and Ogongo based on soil sample analysis.

Soil layer	Mashare				Ogongo			
	1	2	3	4	1	2	3	4
Soil layer depth (m)	0.20	0.40	0.60	1.00	0.20	0.30	0.50	0.80
Bulk density (t/m^3^)	1.51	1.53	1.54	–	1.55	1.67	1.61	–
Soil water content at wilting point (m/m)	0.05	0.06	0.08	–	0.03	0.02	0.02	–
Soil water content at field capacity (m/m)	0.14	0.15	0.17	–	0.09	0.08	0.09	–
Sand content (%)	87.83	87.15	85.62	84.56	89.35	87.62	88.02	88.28
Silt content (%)	3.22	3.00	2.96	3.10	6.66	6.33	6.48	6.28
Organic N concentration (g/Mg)	0.32	0.24	0.20	0.19	0.21	0.18	0.15	0.14
Soil pH	7.05	7.00	7.10	7.23	6.85	6.73	6.88	6.78
Sum of bases (cmol/kg)	5.23	6.56	7.35	4.60	2.17	1.83	2.39	2.80
Organic carbon concentration (%)	0.29	0.20	0.15	0.14	0.18	0.16	0.11	0.09
Calcium carbonate content (%)	0.11	0.27	0.34	0.41	0.04	0.00	0.02	0.06
Soluble NO_3_ concentration (g/Mg)	21.15	8.20	4.04	4.99	5.42	4.46	2.22	4.44
Electrical conductivity (mmho/cm)	0.03	0.02	0.02	0.02	0.02	0.01	0.02	0.02

### Calibration procedure

We calibrated two cowpea parameter sets: cowpea with inoculant and cowpea without inoculant. We started the calibration with the non-inoculated cowpea as a baseline. As the starting set of crop parameter values, we took the standard cowpea crop parameter set in the EPIC crop database file. We limited the parameters available for calibration to the growth-related ones and excluded other non-relevant parameters such as seed cost, price for yield, frost damage or crop category. The list of calibrated parameters is provided in Table 2. We started the calibration by running the model once for each of the two sites. The absolute difference between simulated yields and the median of the reported yields were calculated for both sites and then summated. We chose the median of the measured yields due to the large yield range (Table 3). In a second step, a new set of crop parameter values was proposed based on the random sampling of a Gaussian distribution of possible values generated for each crop parameter. We did not allow the new values to deviate more than 20% from the original values, or the values set down as a feasible range in the EPIC user guide. The model was rerun using the new crop parameter set. If the sum of the differences in yields was lower with this new parameter set, the set was accepted as the new parameter set; if not, the set was rejected and the old one retained. This procedure was iterated 10,000 times. Once the calibration for the non-inoculated cowpea was finished, we repeated the same steps for the inoculated cowpea but started with the final parameter set of the non-inoculated one as the starting set. The reasoning was that we first wanted to calibrate the crop parameters to match the Lutembwe variety and then calibrate the Lutembwe crop parameter set to match inoculated Lutembwe cowpeas. The final parameter values for inoculated and non-inoculated cowpeas are provided in Table 2.

**Table 2:** List of calibrated crop parameters with units, the original values for cowpeas, and the calibrated values for cowpeas without (w/o) inoculum and with inoculum.

Calibrated crop parameters	Unit	Original	w/o inoculum	With inoculum
Radiation use efficiency	–	25.00	20.00	23.72
Harvest index	–	0.45	0.36	0.43
Optimal temperature	°C	25.0	30.0	24.5
Base temperature	°C	12.00	12.73	10.18
Maximum potential LAI	–	5.00	4.02	4.82
Fraction growing season when LAI declines	–	0.75	0.60	0.52
LAI decline parameter	–	1.00	1.03	1.15
Biomass-energy ratio decline parameter	–	1.00	1.20	1.07
Maximum stomatal conductance	m/s	0.0100	0.0109	0.0087
Maximum crop height	M	1.20	1.28	1.36
Maximum root depth	M	1.00	1.08	0.92
Lower limit of harvest index	–	0.05	0.05	0.06
N uptake parameter 1	–	0.0515	0.0412	0.0360
N uptake parameter 2	–	0.0335	0.0300	0.0276
N uptake parameter 3	–	0.0296	0.0299	0.0272
P uptake parameter 1	–	0.0074	0.0074	0.0074
P uptake parameter 2	–	0.0037	0.0037	0.0037
P uptake parameter 3	–	0.0035	0.0035	0.0035
K uptake parameter 1	–	0.0140	0.0160	0.0128
K uptake parameter 2	–	0.0130	0.0111	0.0091
K uptake parameter 3	–	0.0120	0.0115	0.0092
Parameter relating vapor pressure deficit to radiation use efficiency	–	7	8	6
Fraction of root weight at emergence	–	0.40	0.41	0.33
Fraction of root weight at maturity	–	0.20	0.19	0.17
Heat units required for germination	DD	100	100	80
Yield decrease with salinity increase	mmho/cm	0.12	0.12	0.10

**Table 3:** Cowpea yields in t/ha for the variety Lutembwe measured on the different plots; and yield simulated after calibration ("simulated").

Site	Inoculum	Plot 1	Plot 2	Plot 3	Plot 4	Mean	Median	Simulated
Mashare	Without	2.52	1.92	1.83	1.64	1.98	1.87	1.86
	With	3.04	2.12	2.98	2.76	2.72	2.87	2.87
Ogongo	Without	1.08	0.13	1.40	0.95	0.89	1.01	1.10
	With	1.23	2.07	2.30	2.10	1.93	2.09	1.82

### Climate data

To study the potential benefits of using seeds with inoculant, we downloaded climate data for both sites from the ISIMIP database with a spatial resolution of 0.5°. The ISIMIP3b data is based on output of phase 6 of the Coupled Model Intercomparison Project^31^ and includes the five general circulation models GFDL-ESM4, IPSL-CM6A-LR, MPI-ESM1-2-HR, MRI-ESM2-0 and UKESM1-0-LL. The projections on future climate conditions were driven by the three combinations ssp126, ssp370, and ssp585 of relative concentration pathway (RCP) and shared socio-economic pathway (SSP) scenarios. We used the bias-corrected climate data provided in ISIMIP3BASD v2.5^32^.

We analyzed the data for both sites to determine which long-term trends may be expected (for figures, see electronic supplementary material). In Mashare, the mean decadal rise in temperature ranges from 0.01 to 0.18 °C in SSP126, to 0.40–0.77 °C in SSP370, to 0.45–1.05 °C in SSP585. Except for two models in SSP126 (GFDL-ESM4, MRI-ESM2-0), all trends are highly significant. In Ogongo, the mean decadal rise in temperature ranges from 0.02 to 0.18 °C in SSP126, to 0.45–0.77 °C in SSP370, to 0.51–1.01 °C in SSP585. All trends are highly significant except for three models in SSP126 (GFDL-ESM4, MRI-ESM2-0, MPI-ESM1-2-HR). To check for changes in the variability of mean annual temperatures, we calculated the variance for the periods 2015–2040 and 2075–2100. In Mashare, temperature variance increases from the first to the second period in nearly all projections for SSP370 and SSP585 but decreases in four out of five models in SSP126. In Ogongo, temperature variance follows a nearly identical trend as in Mashare: it increases in nearly all projections for SSP370 and SSP585 but decreases in four out of five models in SSP126. Based on these numbers, the projected mean annual temperatures will increase significantly from 2015 to 2100 in almost all scenarios.

Regarding precipitation, there is no clear trend in changes over time in Mashare. In SSP126, mean annual precipitation sums remain more or less constant, without any significant trends up or down. In SSP370 and SSP585, most models project a slight decrease in precipitation, but the trend is only significant in one instance (SSP585/GFDL-ESM4, p = 0.025). The same pattern repeats in Ogongo, with the only difference that the negative trend is significant in two instances (SSP370/GFDL-ESM4 and SSP585/GFDL-ESM4, p = 0.02 and p = 0.007, respectively). To check for changes in the variability of annual precipitation sums, we calculated the variance for the periods 2015–2040 and 2075–2100. In Mashare, precipitation variance decreases in four out of five models for SSP126 and SSP585 and increases in three out of five models for SSP370. In Ogongo, the change in precipitation variance is slightly different; it decreases in four out of five models for SSP126 and increases in three out of five models in SSP370 and SSP585. Based on these numbers, the projected patterns in annual precipitation sums will stay more or less the same from 2015 to 2100 in almost all scenarios.

For the study, we combined the climate data for both sites to have a larger pool of different climatic conditions and classified the data into six categories of annual precipitation sums and mean annual temperatures. We considered using the mean temperature of the cowpea growing season instead of mean annual temperature, but the average difference between mean annual and mean seasonal temperature is only 0.7 °C, so that we stayed with the annual value.

### Model setup for simulation study

For the study, we simulated cowpea cultivation under the same management as in the calibration but did not allow irrigation, as we wanted to examine the performance of the inoculated and non-inoculated cowpeas under water-stressed conditions. For both Mashare and Ogongo, we ran the model for 80 years from 2020 to 2100 and repeated the simulations for 15 combinations of five GCM models and three climate change scenarios. Together, 1200 single estimates of annual yields were thus created for each site, 2400 in total. We analyzed the results jointly for both sites to obtain a larger data pool with different climatic conditions.

### Ethics declarations

The present study was performed in compliance with relevant institutional, national, and international guidelines and legislation.

## Results

### Calibration

We calibrated the cowpea parameter sets to match the median of the yields reported for the four plots allocated to each treatment/variety (Table 3). In the treatment without inoculant, the measured median cowpea yields and the simulated yields of the Lutembwe variety were 1.87 vs. 1.86 t/ha in Mashare; and 1.01 vs. 1.10 t/ha in Ogongo. In the treatment with a heat resistant *Bradyrhizobium* inoculant, the measured median cowpea yields and the simulated yields of the Lutembwe variety were 2.87 vs. 2.87 t/ha in Mashare and 2.09 vs. 1.82 t/ha in Ogongo. The results show that the calibration was successful and that the average yield increase of approximately 1 t/ha due to the treatment with the *Bradyrhizobium* strain could be replicated. The calibration was slightly less successful in Ogongo than in Mashare, but even there the simulated yields fall into the range of reported yields.

### Under which climatic conditions is it most beneficial to switch to inoculated cowpeas to increase yields?

**Figure 1:**
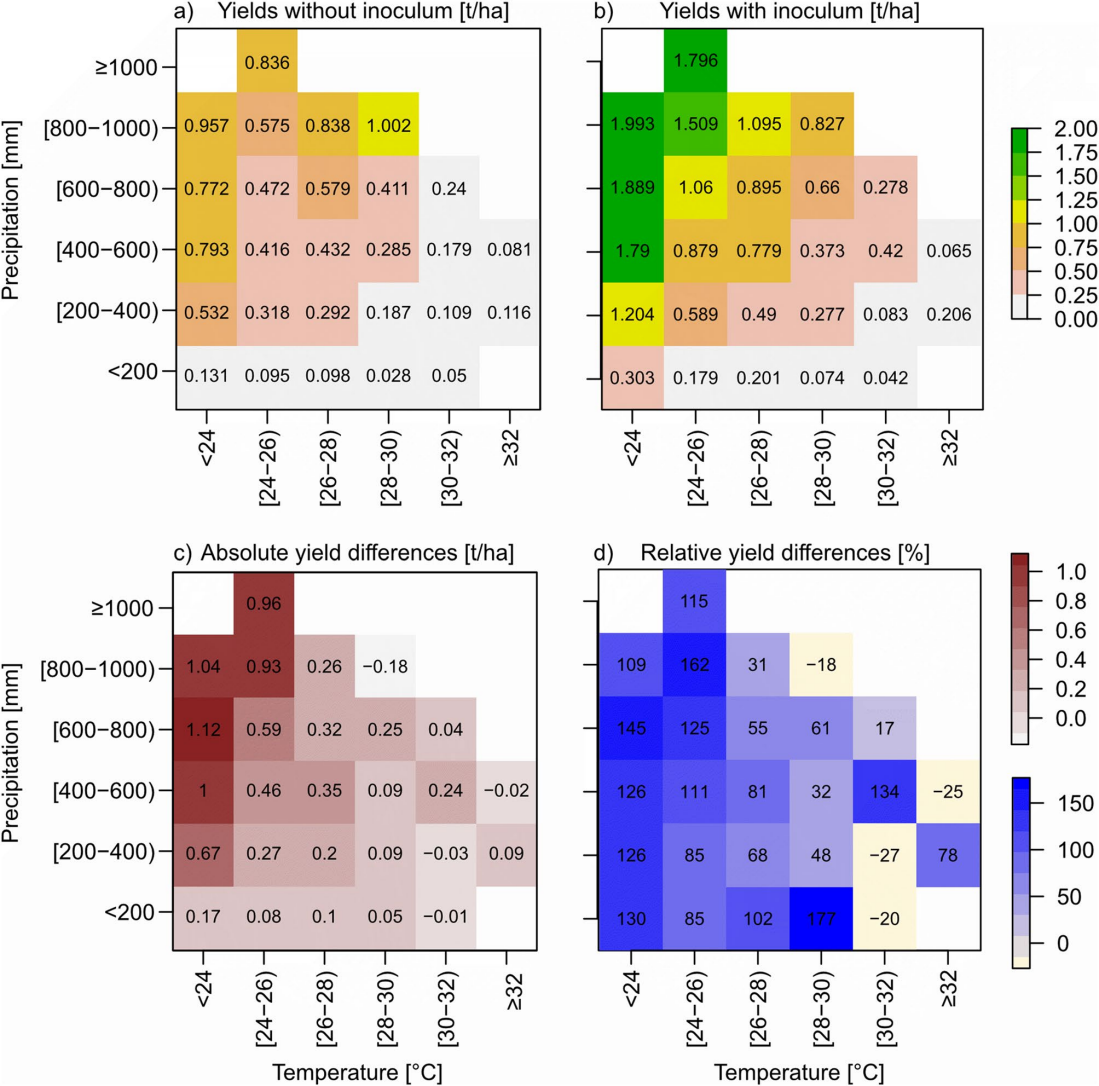
Cowpea yields without (**a**) and with (**b**) inoculant produced in years with specific annual precipitation sums (y-axes) and mean annual temperatures (x-axes); the second row shows absolute (**c**) and relative (**d**) differences between the yields plotted in (**a**) and (**b**), with (**a**) as the baseline.

We simulated yields of cowpeas with and without inoculum. Over the whole dataset, mean yields of cowpeas without inoculum are 0.47 t/ha (min = 0.001 t/ha, max = 2.51 t/ha, sd = 0.54 t/ha), and mean yields of cowpeas with inoculum are 0.99 t/ha (min = 0.001 t/ha, max = 3.66 t/ha, sd = 0.90 t/ha). The differences between the cowpea yields with and without inoculum are significant (*p*-value = 0.000). The highest yields for cowpeas without inoculum are produced in years where annual mean temperatures are below 24 °C with precipitation higher than 400 mm, and in years where precipitation exceeds 800 mm, regardless of temperature. In these years, simulated yields range from 0.58 to 1.0 t/ha (Fig. 1a). The lowest yields (0.03 to 0.18 t/ha) occur in years with mean temperatures higher than 30 °C and in years with precipitation below 200 mm. Yields of cowpeas with inoculum follow a similar pattern (Fig. 1b), but here the highest yields range from 0.8 to 1.99 t/ha, and the lowest from 0.04 to 0.2 t/ha, with the very low yields only starting after mean temperatures rise above 32 °C.

In terms of absolute differences, the highest yield benefits occur in years with temperatures below 24 °C and precipitation above 200 mm, and in years with temperatures up to 26 °C and precipitation higher than 800 mm (Fig. 1c). They range from 0.67 to 1.12 t/ha. In terms of relative differences, the picture is a little different and not as clear (Fig. 1d). High relative yield increases can be achieved in years where temperatures are below 26 °C, regardless of precipitation, but also in years where precipitation is below 200 mm and temperatures below 30 °C. In these years, yield increases of up to 177% are attainable by switching to inoculated seeds. Slightly lower yield increases of 31–102% can be achieved in years with average temperatures of 26–28 °C, regardless of precipitation. However, the yields in some of these years are so low that an increase of 61% can mean an absolute increase of 0.1 t/ha. The additional cost of inoculated seeds would thus likely not pay off in these years. Interestingly, there are also years where using inoculated cowpeas would lead to lower yields. These instances can be observed mainly in dryer years with temperatures above 30 °C. However, cowpea yields in these years are close to zero, so that the negative effect may seem large but is negligible.

Based on the simulations, it can be concluded that inoculated cowpeas provide yield advantages mainly in years where growing conditions are favorable, i.e., colder and wetter years. They also have a slightly higher drought tolerance than non-inoculated cowpeas, but the yield difference is very small. Non-inoculated cowpeas, on the other hand, have a slightly higher tolerance for high temperatures, but again, the yield difference is marginal.

### Can inoculated cowpeas withstand water stress better than non-inoculated plants?

**Figure 2:**
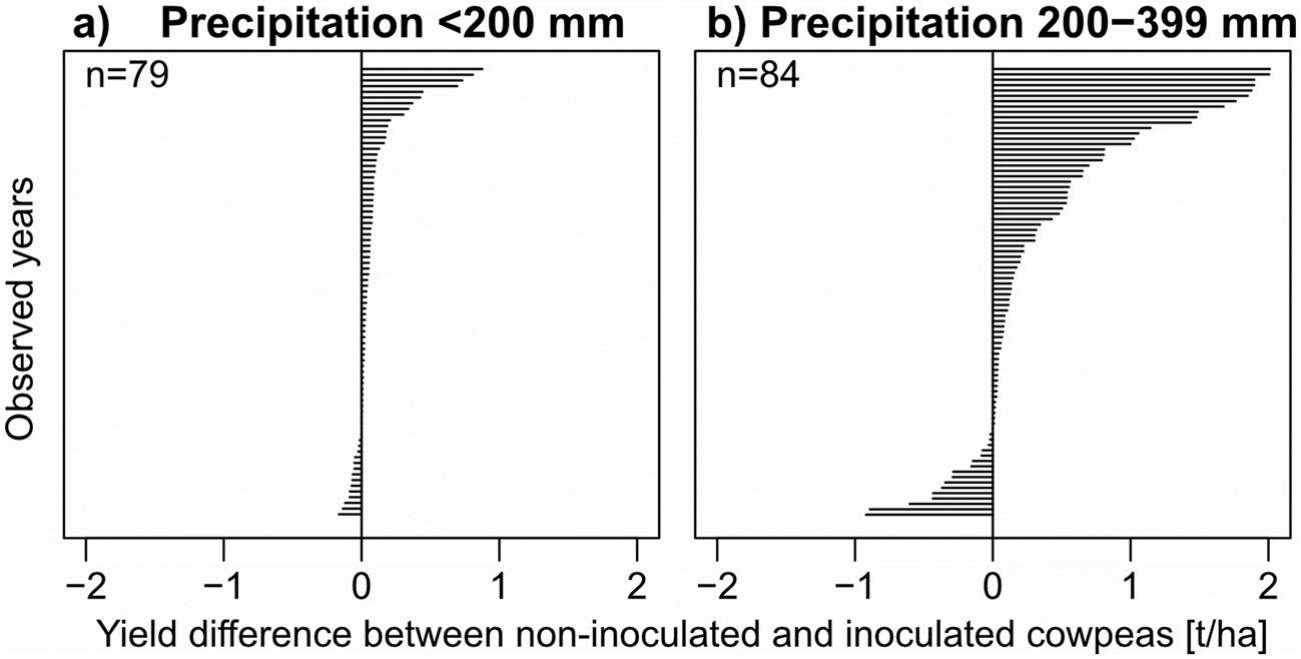
Yield differences between inoculated and non-inoculated cowpeas in years where precipitation is (**a**) below 200 mm and (**b**) between 201 and 400 mm. Negative values indicate that non-inoculated cowpeas had higher yields than inoculated ones, positive values indicate the opposite.

On average, inoculated cowpeas have slightly higher yields than non-inoculated cowpeas in very dry years, as long as temperatures stay below 30 °C (Fig. 1). If all instances of years with precipitation below 200 mm are considered, not only the average, yield differences range between -0.16 and 0.88 t/ha (Fig. 2a), with a median of 0.04 and a mean of 0.09 t/ha. Even though the differences are small, they are highly significant (p = 0.000). This shows that in years with dry conditions, there are yield advantages associated with using inoculated cowpeas, but in most instances, the difference is very small. If the year is slightly wetter, with precipitation sums of 200–400 mm, the yield advantage is increasing (Fig. 2b, with a range of yield differences from -0.92 to 2.01 t/ha, a median of 0.13 and a mean of 0.37 t/ha). In these years, the yield advantage is thus more pronounced. The conclusion is that while inoculated cowpeas do have a significantly higher yield than non-inoculated cowpeas under dry conditions, farmers may only markedly benefit from using them in years with a little more precipitation.

### Will using inoculated cowpeas yield the same benefits in the future as they do now?

**Figure 3:**
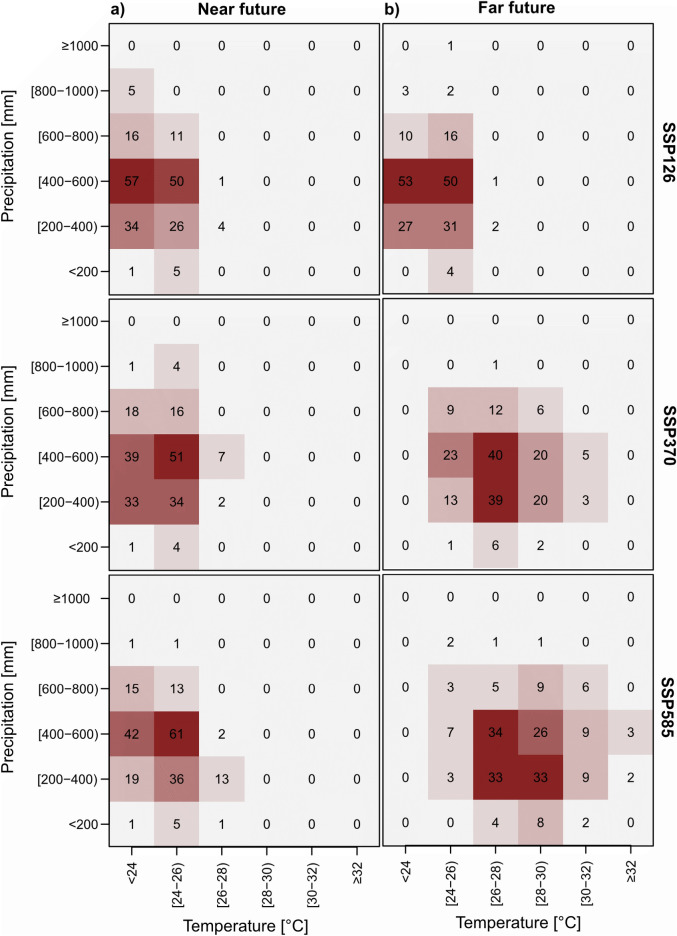
Change in precipitation and temperature from (**a**) the near (2030–2050) to (**b**) the far (2080–2100) future for SSPs 126, 370, and 585. The total data pool for each grid consists of 210 observations: 21 years, five GCM models and two sites (Ogongo, Mashare). Each raster cell shows the number of instances observed for the combination of temperature and precipitation class.

Figure 3 shows the change in climatic conditions from the near (2030–2050) to the far (2080–2100) future based. In scenario SSP126, most years have climatic conditions with annual precipitation sums between 400 and 600 mm and mean annual temperatures of < 24 °C and 24–26 °C in both the near and the far future (Fig. 3, first row). In SSP370, most years have a precipitation of 400–600 mm and a temperature between 24 and 26 °C in the near future. There are more years with a precipitation of only 200–400 mm than in SSP126. In the far future, temperatures rise and precipitation sums decrease and most observations fall into the temperature class 26–28 °C and the precipitation classes 200–400 and 400–600 mm (Fig. 3, second row). In SSP585 in the near future, conditions are similar to SSP370, but in the far future, there are more years with higher temperatures of 30–32 °C and even years with mean annual temperatures of > 32 °C (Fig. 3, last row).

If we take the frequencies with which climate conditions occur in each of the scenarios as weights and calculate a weighted mean of yields over all climate conditions, we see that in the near future (2030–2050), a yield increase of 0.56–0.63 t/ha can be achieved by using inoculated cowpeas (Table 4). The yield difference stays roughly the same in the far future for SSP126 but decreases in the other two climate scenario to 0.23–0.29 t/ha. The conclusion is that given the frequency with which certain climatic conditions may occur in the near and far future, using inoculated cowpea seeds instead of non-inoculated ones may only be a reasonable alternative in the near but not in the far future.

**Table 4:** Weighted means of cowpea yields (t/ha) with and without (w/o) inoculum in the near and far future under three different climate change scenarios.

Time period	Climate data	Yields w/o inoc	Yields with inoc	Yield difference
Reference (2017–2018)	SASSCAL WeatherNet	0.47	0.99	0.52
Near future (2030–2050)	SSP126	0.48	1.11	0.63
	SSP370	0.48	1.04	0.56
	SSP585	0.48	1.11	0.63
Far future (2080–2100)	SSP126	0.45	1.10	0.65
	SSP370	0.46	0.75	0.29
	SSP585	0.41	0.64	0.23

The weights are the frequencies with which climate conditions occur in each scenario (Fig. 2).

## Discussion

Cowpeas are an important staple food crop and protein source for many subsistence farmers in sub-Saharan Africa. Due to their symbiosis with N_2_-fixing bacteria, they require no or only low doses of additional nitrogen fertilizer. However, the yield and N_2_-fixation are strongly affected by stresses, especially water stress^19^. To enhance the positive properties of legumes even under stressed conditions, their inoculation with rhizobia has become the most used technique^33^, whose popularity has increased even further in recent years^34^. For several different legumes it has been shown that nitrogen-fixing root nodule symbiosis can enhance drought tolerance of the plants^35–38^.

In an experiment in the savannah zone of Nigeria, yield increases of 27–33% could be observed for soybeans that had been inoculated^39^. In our study, cowpea yields could be increased on average by 47%, but with a high variation across climatic conditions. A recent review of the technique thus concluded that rhizobial bioinoculants could play an important role in next-generation agriculture, especially soil fertility management; but only if elite strains that combine competitiveness and effectiveness in field conditions are isolated and used^40^. This means that the strains should be able to compete effectively with native rhizobia for nodule occupancy, have a high nitrogen fixation ability, be stress tolerant, have genetic stability in the manufacturing process, and possess limited year-to-year persistence in unplanted soils so that they can be replaced without problems if improved strains are introduced in the future^41^.

However, even if high-performing strains are isolated that meet all these conditions, there is still no assurance that they will perform well under all conditions or that farmers can gain a financial advantage by using them. In our study, yield differences between inoculated and non-inoculated cowpeas strongly varied between years and mainly depended on the climatic conditions. Inoculated cowpeas yielded an advantage mainly in years with favorable climatic conditions; in dry or hot years, yield differences were negligible. A study in Kenya examining inoculated and non-inoculated soybeans also found a considerable variation in yields, from 0 to 2.6 t/ha, with an average of 0.6 t/ha^39^. In this study, the authors performed a simple financial analysis: they estimated that for planting one hectare, 80 kg of soybean seeds were required, for which 320 g of peat-based soybean inoculant is needed, at a cost of US$ 13.4. At the time of the analysis, soybeans in Kenya were sold at about US$ 0.5 per kg, which means that yields needed to be increased by at least 27 kg/ha to recover the cost of the improved seeds. For cowpeas, approximately 20 kg/ha of seeds are required. The price for one kg of cowpeas was about US$ 0.8 in Namibia in 2021^42^. If we assume the same costs for cowpea inoculants as for soybean inoculants, and that the same amount of inoculum is required for cowpeas as for soybeans, it means that cowpea yields need to increase by only 4.1 kg/ha for farmers to recover the cost of the inoculated seeds. Our study shows that this increase can be achieved in nearly all years, except some years with annual mean temperatures higher 30 °C, and thus that using inoculated cowpea seeds can benefit subsistence farmers in Namibia. However, it may be difficult for farmers to purchase the product, as there is currently only one manufacturer in Africa (Kenya) that produces rhizobia inoculants for cowpeas^40^; the other manufacturers mainly sell strains for soybeans. Generally, cowpeas are still an underexploited crop, and applied plant breeding can likely lead to large gains for small investments^3^. It is also promising that recently new *Bradyrhizobium* strains and species have been isolated in Namibia that are highly temperature resistant in comparison to many other bradyrhizobia^43^.

The study we present in this paper also shows that using crop models to augment the scope of field experiments can considerably increase the amount of information that can be learned. In crop modeling, data from field experiments are mainly used to test and validate the ability of models to predict the effects of different management options on factors such as yields, soil dynamics, or emissions (e.g.^44,45^). Once models have been sufficiently tested and validated, they can be used to determine potential causes of observed dynamics in field experiments (e.g.^46^) or upscale field experiments in time and space (e.g.^47–49^). In our study, we assume that the long-established crop model EPIC has been sufficiently tested and validated in the past and can be applied to the issue without further testing. We used the data collected in the field trials to calibrate the model and then applied it to upscale the field experiment to include many more variations of climatic conditions than could be observed in the actual trial.

In conclusion, our study showed that non-inoculated cowpeas have significantly lower yields than inoculated cowpeas averaging 0.5 t/ha. If climatic conditions are favorable (cool and wet), yield differences can increase to over 1 t/ha. In dry years (< 200 mm), the average yield difference decreases to only 0.1 t/ha. In the far future (2080–2100), instances of dry and hot years will increase, so that using inoculated cowpea seeds instead of non-inoculated ones may not benefit farmers as much as in the present and near future (2030–2050). We conclude that using cowpea seeds inoculated with an efficient rhizobia strain can significantly increase yields under varying climatic conditions, but that yield advantages decrease markedly in very dry and very hot years.

## Supplementary Information

Supplementary Information.

## Data Availability

The datasets generated during and/or analyzed during the current study are available from the corresponding author on reasonable request.
