# Supplementary material for: Exploring the benefits of inoculated cowpeas under different climatic conditions in Namibia
Source: Sci Rep. 2023 Jul 20;13:11761. doi: 10.1038/s41598-023-38949-2 (PMC10359254; doi:10.1038/s41598-023-38949-2)
Supplement: Supplementary file 1 — Supplementary Information. [file 41598_2023_38949_MOESM1_ESM.pdf]

Supplementary materials for the paper:

Exploring the benefits of inoculated cowpeas under different climatic conditions in Namibia

Livia Rasche[1*], Joscha N. Becker[2], Percy Chimwamurombe[3], Annette Eschenbach[2], Alexander Gröngröft[2,3], Jihye Jeong[1], Jona Luther-Mosebach[2], Barbara Reinhold-Hurek[4], Abhijit Sarkar[4], Uwe A. Schneider[1]

[1] Universität Hamburg, Research Unit Sustainability and Climate Risks, Grindelberg 5, 20144 Hamburg, Germany
[2] Universität Hamburg, Institute of Soil Science, Allende-Platz 2, 20146 Hamburg, Germany
[3] Namibia University of Science and Technology, Department of Natural and Applied Sciences, Brahms St, Windhoek, Namibia
[4] University of Bremen, Research Group Molecular Plant-Microbe Interactions, Loebener Str. 5, 28359 Bremen, Germany

Mashare

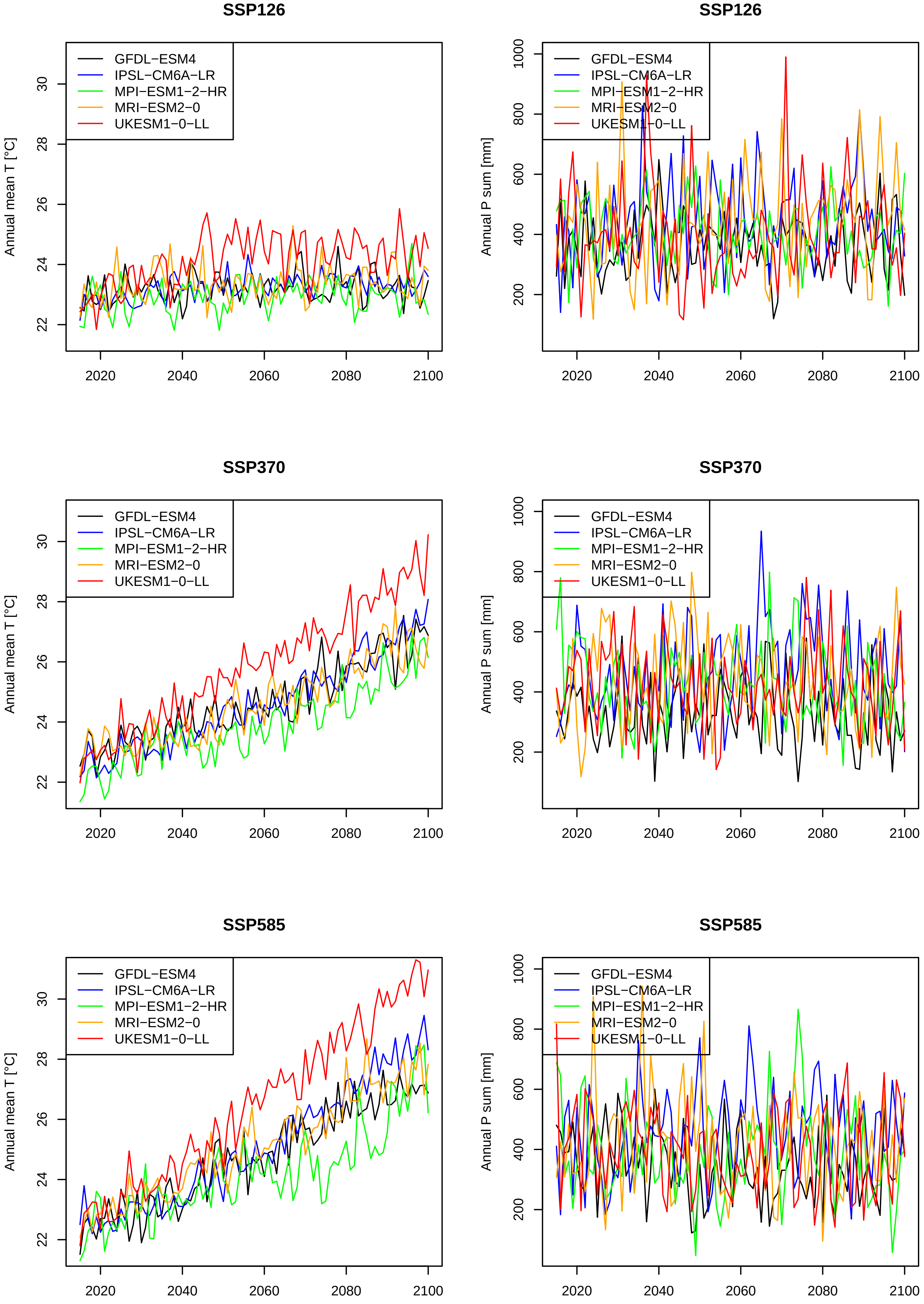
SSP126
SSP370
SSP585
GFDL−ESM4
IPSL−CM6A−LR
MPI−ESM1−2−HR
MRI−ESM2−0
UKESM1−0−LL
Annual mean T [°C]
Annual P sum [mm]
2020
2040
2060
2080
2100

Ogongo

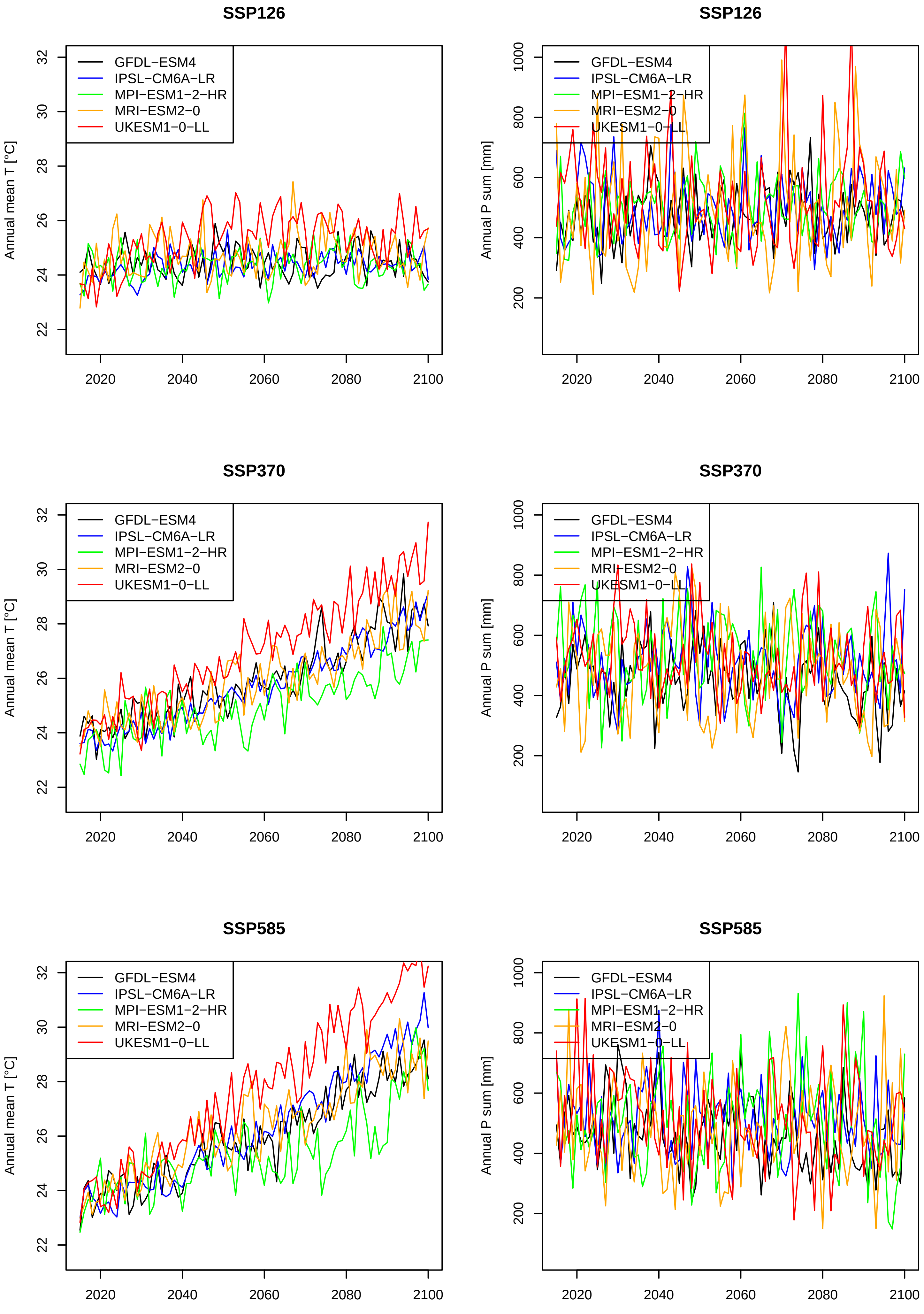
SSP126
SSP126
SSP370
SSP370
SSP585
SSP585
Annual mean T [°C]
Annual P sum [mm]
GFDL−ESM4
IPSL−CM6A−LR
MPI−ESM1−2−HR
MRI−ESM2−0
UKESM1−0−LL
22
24
26
28
30
32
200
400
600
800
1000
2020
2040
2060
2080
2100
